# Evaluation of the Effect of Dexmedetomidine on Postoperative Cognitive Dysfunction through Aβ and Cytokines Analysis

**DOI:** 10.22037/ijpr.2020.113576.14381

**Published:** 2021

**Authors:** Zhi Li, Shanglong Yao, Minghua Cheng, Jianyan Chen

**Affiliations:** a *Shantou University Medical College, Shantou, China. *; b *Department of Anesthesiology, Second People’s Hospital of Futian District, Shenzhen, China. *; c *Department of Anesthesiology, Union Hospital, Tongji Medical College, Huazhong University of Science and Technology, Wuhan, China. *; d *Department of Anesthesiology, The First Affiliated Hospital of Shantou University Medical College, Shantou, China.*; e *Department of Anesthesiology, The First Affiliated Hospital of Guangdong Pharmaceutical University, Guangzhou, China.*

**Keywords:** Dexmedetomidine, Postoperative cognitive dysfunction, β-amyloid, Cytokine, Elderly

## Abstract

Postoperative cognitive dysfunction is a common postoperative neurological complication in elderly patients, and has some relationship with neuroinflammation. some studies have shown ability of dexmedetomidine to improve cognitive performance in elderly individuals who underwent thoracic surgery. Therefore, our study hypothesized that dexmedetomidine treatment may reduce the incidence of POCD in elderly patients.In addition,this study detected the antineuroinflammatory effects of dexmedetomidine by β-amyloid aggregation inhibitors and release of cytokines in elderly patients . The results show that dexmedetomidine used during operation can inhibit the postoperative release of Aβ and cytokines in elderly patients, and dexmedetomidine used during operation can reduce the incidence of postoperative cognitive dysfunction, with dose-dependence. These results provide a clinical application direction for clinical anesthesiologists and ICU physicians.

## Introduction

Postoperative cognitive dysfunction (POCD), characterized by mental derangement, anxiety, personality changes, and memory impairment is a common postoperative neurological complication in the elderly patients (1). 

POCD often lead to a longer hospital stay, a decline in life quality, and an increase in morbidity and mortality rates (2). More importantly, this short-term dysfunction might cause a permanent cognitive impairment, such as Alzheimer’s disease (AD), which means the patient might lose their independent ability of daily life and causes serious post-operation physical and psychological damage (3). Previous studies have suggested that age, the surgery itself and anesthesia may be the possible causes of POCD (4, 5). With the rapid increase in the aging population in the world, the amount of surgery in elderly patients also increased tremendously; Therefore, POCD has become a major concern for the anesthetis (6).

Although the risk factors as mentioned earlier were known, the exact mechanism of POCD is still not fully understood. In the past, studies on POCD mainly focused on commonly recognized pathological changes in nerve cells, such as the presence of senile plaques (SP) (7) and changes in the cerebral cortex and hippocampus (8) Recently, researchers have begun to pay attention to internal changes of neuroinflammation existing in nerve cells (7), such as β-amyloid (Aβ) protein (9), IL-1β (10), and IL-6 (11) because they accurately reﬂect brain damage in operation and are closely related to postoperative cognitive function. It has been reported that accumulated Aβ triggers NLRP3 inﬂammasome in microglial cells, resulting in an increased release of cytokines such as TNF-𝛼, IL-1β and IL-6 (12). 

Dexmedetomidine is a highly selective and potent alpha 2-adrenoreceptor agonist, providing excellent sedation and analgesia. It may cause significant hypotension and bradycardia but has minimal effect on respiratory drive (13). In the present study, a growing number of studies have revealed that dexmedetomidine significantly improved neuron viability *in-vitro *model of AD (14). In addition, some studies have shown its ability to improve cognitive performance in elderly individuals who underwent thoracic surgery (15). Therefore, we hypothesized that dexmedetomidine reduces the incidence of POCD in elderly patients.

To validate the hypothesis, the cognitive function and the incidence of POCD were investigated via intravenous administration of dexmedetomidine, which was used complementary to the general anesthesia at different doses in elderly patients undergoing spine surgery. Furthermore, our study determined the serum levels of β-amyloid and inflammatory cytokines in elderly patients undergoing spine surgery at different doses of dexmedetomidine, attempted to elucidate the mechanism of dexmedetomidine against neuroinflammatory damage.

## Experimental

*Ethics considerations*

The protocol was approved by the Ethics Committee of Baoan Hospital Affiliated to Southern Medical University (Shenzhen, China). The study was conducted in accordance with the guidelines of Good Clinical Practice and the principles expressed in the Declaration of Helsinki. Written informed consent was obtained for each patient.

*Patient recruitment*

Between Oct 2015 and April 2017, one hundred and twenty elderly patients (over 65 years), ASA I-III, were recruited to participate in the study. The patients were randomly assigned equally into four groups to receive normal saline infusion (group C) or dexmedetomidine infusion, including small-dose group (group S), medium-dose group (group M), large-dose group (group L). Patients and researchers were blind to treatment allocation. Exclusion criteria were: i) Patients aged <65 or >90 years; ii) patients with a Mini-Mental State Examination (MMSE) score less than 24; iii) patients with mental and neurological diseases that may affect the level of consciousness; iv) patients with abnormalities in hepatic or renal function; v) patients suffering from preoperative bradycardia or hypotension.

*Procedures*

Patients fasted for 8 h before the operation, and no premedication was given. When the patients entered the operating room, catheterization of the left radial artery and a large vein in the right forearm was performed. All patients received general anesthesia by the same anesthesiologist. The patients in 3 dexmedetomidine groups (groups S, M, and L) were administered DEX with a loading dose of 0.3 mcg/kg over 10 min before induction of anesthesia. They then received the maintenance doses of 0.2, 0.5, and 0.8 mcg/kg/h, respectively, after anesthesia induction The control group received a placebo infusion of normal saline. In all groups, anesthesia was induced with fentanyl (23 mcg/kg) and propofol (1-2 mg/kg). Tracheal intubation was facilitated with 0.15 mg/ kg cisatracurium. Anesthesia was maintained with propofol and remifentanil, cisatracurium intermittently as required. The patients were mechanically ventilated, and the partial pressure of end-tidal carbon dioxide (PetCO_2_) was maintained at 35–45 mm Hg. The depth of anesthesia was monitored and recorded by a Bispectral Index™ (BIS) sensor (BIS monitor Model A2000; Aspect Medical System, Norwood, MA, USA) applied to the forehead. The anesthesia was guided to reach a BIS value of 4060^ (^^16^^)^. Hemodynamic stability was maintained, the variation of mean arterial blood pressure (MAP) from preanesthetic value was kept less than 10%^ (^^17^^)^. Propofol and remifentanil were discontinued 5 min prior to the end of the surgery, while the infusion of dexmedetomidine or normal saline was stopped 40 min prior to the end of surgery. Flurbiprofen axetil combined with fentanyl for postoperative analgesia was administered in all patients, visual analog scale (VAS) scores were controlled less than 3.

*Measurements and blood samples*

During the investigation, HR, electrocardiography (ECG), MAP, SpO2, PetCO_2_ and BIS were continuously monitored. The occurrence of hypotension and bradycardia, time of operation (T_e_), time from the end of surgery to opening eyes (T_w_) were also recorded.

Cognitive function was evaluated with the Mini-Mental State Examination (MMSE) immediately before the induction of anesthesia (D_0_), and day 1(D_1_), day 3(D_2_), and day 7(D_3_) after the operation. Compared with baseline, postoperative dysfunction was considered according to the criteria of one standard deviation of a certain test item being reduced at least. When postoperative dysfunction happened on two or more test items of a patient, then the patient suffered POCD. 

Venous blood (10 mL at each time point) was drawn to determine the concentrations of β-amyloid, TNF-α, IL-1β and IL-6. Blood samples were drawn via an indwelling catheter inserted into the forearm vein and put into heparin anticoagulant tubes, transported to the hospital research laboratory within 30 min, and then centrifuged at 2,000 ×g for 10 min at 4 °C. Separated plasma samples were stored at -80 °C until assayed. Using commercially available ELISA kits, plasma levels of β-amyloid, TNF-α, IL-1β and IL-6 were measured using the ELISA technique. Human β-amyloid ELISA Kit and Human TNF-α, IL-1β, IL-6 ELISA Kit were obtained from Thermo Fisher Scientific Co., Ltd (Boston, USA). 

*Statistical analysis*

SPSS version 13.0 (SPSS Inc., Chicago, IL, USA) was used to perform the statistical analysis for our study. Quantitative variables are presented as mean ± standard deviation or median with interquartile range. Categorical variables were presented with the number of cases (%). The t-test was used to compare the statistical diﬀerence between 2 groups. One-way ANOVA and the least signiﬁcant diﬀerence (LSD) were employed to compare the expression level of proteins at diﬀerent time points in the same group. Nominal data were analyzed using χ2 or Fisher’s exact tests. *P* < 0.05 was considered statistically significant. 

## Results

*Demographic and clinical features*

As shown in Table 1, general demographic diﬀerences about age, sex ratio, height, weight, ASA grade, operation time between the four groups of patients were not statistically signiﬁcant.

*Evaluation of the cognitive function during the perioperative period* **.**

Table 2 illustrates the preoperative MMSE scores. Compared with preoperation, the MMSE scores at day 1, day 3 and day 7 after surgery were significantly decreased (*P* < 0.01) in group C and group S, while no significant difference was observed in group M and group L. Compared with the control group, the MMSE scores at day 1, day 3 and day 7 after surgery were significantly higher in group M and group L(*P* < 0.01). In contrast, no significant difference was observed in group S.

*Incidence of POCD after surgery*

Table 3 illustrates the incidence of POCD in the four groups after surgery. Thirteen patients in the control group and 11 patients in the group S developed POCD. There was no significant difference between the two groups (*P* = 0.598). Compared with the control group, the number of patients who developed POCD in group M (5 patients) and group L (3 patients) decreased significantly (*P* < 0.05, *P* < 0.01, respectively), but there was no significant difference between the 2 groups. 

*Incidence of adverse effects and recovery time*

As shown in Table 4, the incidence of hypotension and bradycardia were the highest in group L (*P* < 0.01), which also had a longer recovery time (Tw) than the other 3 groups (*P* < 0.05).

*Serum levels of β-amyloid, TNF-α, L1β and IL6*

**Figure 1:**
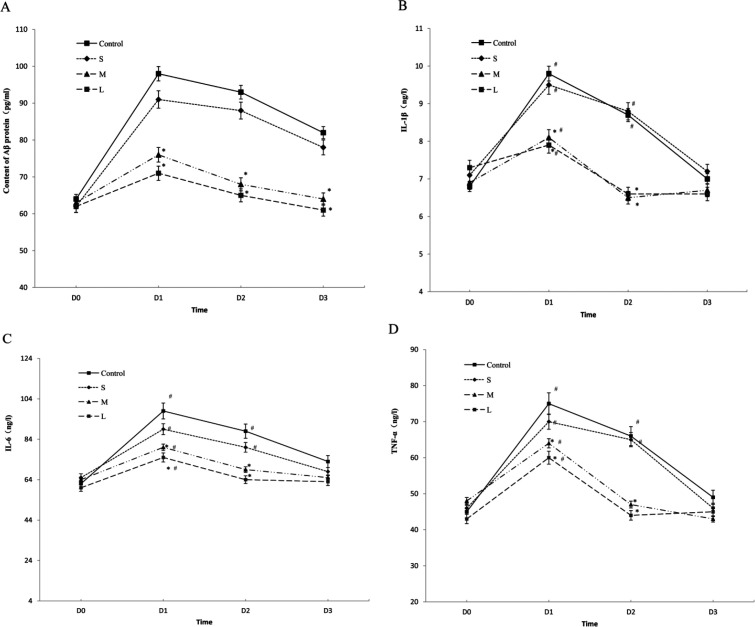
Serum levels of β-amyloid, TNF-α, IL‑1β and IL‑6. The concentrations of  -amyloid (A), inflammatory cytokines including IL-1  (B) ,IL-6 (C), and TNF-α (D) were determined in elderly patients undergoing* spine* *surgery* at different doses of DEX. at different time. ^*^P < 0.05 *vs.* before surgery; ^#^*p *< 0.05 *vs.* control group. DEX: Dexmedetomidine

Compared with baseline values (preope-ration), Figure 1 shows the serum levels of β-amyloid were markedly increased at day 1 after the operation in all the groups, then showed a gradual decrease, while at day 7 after the operation, the levels of β-amyloid decreased to normal as compared to the preoperative level in the group M and L (*P* = 0.122), but the control group and the S group still had a signiﬁcantly higher level of β-amyloid at day 7 after the operation (Figure 1A, *P *< 0.01). 

Compared with baseline values (preoperation), Figure 1 shows the serum levels of TNF-α, L1β and IL6 were markedly increased at day 1 after the operation in all the groups, then showed a gradual decrease, while at day 3 after the operation , the levels of TNF-α, L1β and IL6 decreased to normal as compared to the preoperative level in the group M and L (*P* = 0.122). However, in the control group and the S group, the serum levels of TNF-α, L1β and IL6 remained a signiﬁcantly higher level at day 3(*P *< 0.01), then decreased to normal at day 7 after the operation (Figures 1B-1D, *P* > 0.05). 

Overall, Compared with the control group，the serum levels of β-amyloid, TNF-α, L1β and IL6 were signiﬁcantly lower in the group M and L (*P* < 0.01) at time points (day 1, day 3 after the operation) with a pairwise comparison, while there was no significant difference in the group S (*P* > 0.05).

## Discussion

The aggravating trend of the aging population brings the incidence and severity of POCD. The incidence of POCD in elderly patients is very high. Tan CB *et al.* reported that the average incidence of POCD was 47% (18). Shoair OA *et al.* showed POCD was observed in 15.9% of older adults even 3 months after major noncardiac surgery (19). In our study, the incidence of POCD in elderly patients after spine surgery was 43% in the control group. The results are consistent with the relative literature.

Although POCD is common, its pathophysiologic mechanism is poorly understood. Anesthesia may also be one of the causes of POCD. General anesthetics, particularly inhaled agents, are likely to be associated with POCD (20). So it is very important to search for safe and effective anesthetics to improve the quality of anesthesia and decrease the incidence of POCD. It has been reported that dexmedetomidine has potential neuroprotective effects *in-vitro * (14). Sato K *et al.* found dexmedetomidine improved neurologic outcome from incomplete ischemia in the rat (21); Taniguchi T *et al. *found that dexmedetomidine could dose-dependently attenuated extremely high mortality rates and increases in plasma cytokine concentrations after endotoxin injection (22).

In our study, For evaluation of the neuroprotective effects *in-vivo*, we used three different doses of dexmedetomidine in elderly patients after spine surgery. Compared with preoperation, our study found that the MMSE score decreased significantly from day 1 to day 7 after surgery in the control group and the small-dose group. Although the incidence of POCD after surgery in the small-dose group decreased, but no significant difference was observed compared with the control group (40% *vs.* 43%, *P* > 0.05). This finding showed that administering DEX with a small dose could not significantly reduce the incidence of POCD after surgery. However, the MMSE scores from day 1 to day 7 after surgery in the medium-dose and large-dose groups had no obvious change compared with preoperation (*P* > 0.05). Compared with the control group, the incidence of POCD after surgery decreased significantly (17% in group M, 13% in group L, respectively). The study demonstrated that the incidence of POCD after surgery was markedly reduced by intravenously administered dexmedetomidine with medium-dose or large-dose. In consideration of the effect of DEX was dose-dependent, we deduced that DEX could exert its anti-inflammatory and neuroprotective effects sufficiently only with the enough concentration. But when administering dexmedetomidine with large dose in elderly patients, the incidence of hypotension and bradycardia was the highest (*P* < 0.01), which also had longer recovery time (Tw) than the other 3 groups (*P* < 0.05), this showed administering dexmedetomidine with large dose for assisting anesthesia was not suitable for elderly patients.

Nowadays, many exciting discoveries have illustrated that neuroinflammation characterized by the release of pro-inflammatory mediators from activated microglia exerts an important effect on the pathophysiology of POCD (23). Microglia cells are a type of macrophage located in the CNS contributing to the inflammatory reactions occurring during POCD pathogenesis by binding to soluble Aβ via cell-surface receptors. The activated microglia cells and astrocytes release inflammatory factors and cytokines such as TNF-𝛼, IL-6, and IL-1β, which invade the immune system, activate the complement system and cause an inflammatory reaction in the central nervous system. It affects the functioning of synaptic connections, and results in cognitive function damage (7). To elucidate the neuroprotective mechanism of dexmedetomidine, we further study the effect of DEX on the production of β-amyloid and the pro-inflammatory factors.

Aβ is one of the normal metabolic products of β-amyloid precursor protein (APP) in the body. A study has suggested that the concentration-dependent dual role of Aβ in neurons is neurotrophic and neurotoxic. Low concentrations of Aβ have been shown to stimulate differentiation of immature neurons. As the concentration rises, Aβ has a neurotoxicity eﬀect, including retraction of dendritic and axonal retraction and a decrease or absence of neuronal cells in mature diﬀerentiated neurons (24). Possible mechanisms of Aβ toxicity in POCD may relate to enhanced oxidative stress, induced apoptosis (25), a systematically inﬂammatory response, synaptic dysfunction, central cholinergic damage, and accelerated phosphorylation of the tau protein (26). Thus, inhibition or reduction of the production and deposition of Aβ is the key to the successful prevention and treatment of POCD.

The results of our study demonstrate that the β-amyloid protein contents increased in all of the groups and reached a peak at 1 day after surgery, which suggests that the factors in the operation and the combined eﬀects of anesthesia may have induced cognitive impairment. Due to the disappearance of surgical stimulation, stress, and other factors, the contents began to drop after surgery were restored to pre-operative levels in the dexmedetomidine group (group M and L) 7 days after surgery. However, the levels were still higher in the control group and group S compared to the situation before surgery. Compared to the control group, the content of β-amyloid protein in the dexmedetomidine group (group M and L) after the operation was signiﬁcantly lower. Our results indicated that dexmedetomidine intervention with medium or high doses could significantly reduce the secretion of β-amyloid and modulate the neuroinflammation associated with surgery and anesthetics.

Our study also showed that the plasma levels of IL1β, IL6 and TNF-α were attenuated by the administration of dexmedetomidine with medium or high dose perioperatively. Compared with the control group, the concentrations of IL1β, IL6 and TNF-α were significantly lower in the dexmedetomidine group (group M and L) at 1 day, 3 days following surgery. These changes were consistent with that of Aβ, indicating that dexmedetomidine administration with medium or high dose during surgery could significantly reduce the inflammatory reaction. The suppression of the production of cytokine may be partially mediated by inhibiting the secretion of Aβ.

However, there have somewhat different changes between Aβ and cytokines. In the dexmedetomidine groups, compared with baseline values, the serum levels of TNF-α, L1β and IL6 decreased to normal three days after the operation, but the serum levels of β-amyloid remained higher level and decreased to normal until seven days after the operation. It has been reported that the formation of the active form of IL-1β is a complex process that typically requires two independent steps with different signals, respectively: induction of pro-IL-1β and activation of caspase-1 (27, 28). In our study, the serum levels of IL1β declined to normal rapidly and preceded the decline of the serum levels of β-amyloid, this indicated that Dexmedetomidine might block both the induction of pro-IL-1β and the activation of caspase-1 by inhibiting the secretion of A𝛽. IL-1β secretion play a crucial role in the pro-inflammatory response, unleash an inflammatory cascade that eventually results in neuronal dysfunction and death. Our study suggested that dexmedetomidine exerted its anti-neuroinflammatory activity by inhibiting the IL-1β secretion at an appropriate dose.

**Table 1:** Demographic and clinic features in the 4 groups

**Characteristics**	**Group S**	**Group M**	**Group L**	**Control group**	***P*** **-value**
Age (years)	74.7 ± 2.6	71.2 ± 3.5	69.8 ± 4.3	73.4 ± 5.1	0.627
Gender (M/F)	17/13	16/14	18/12	17/13	0.819
Weight (kg)	60.3 ± 4.2	61.5 ± 3.7	63.2 ± 5.3	62.4 ± 4.5	0.782
Height (m)	1.67 ± 0.08	1.63 ± 0.11	1.65 ± 0.07	1.69 ± 0.12	0.526
ASA^*^grade (I/II/III)	2/21/7	4/16/10	5/18/7	4/17/9	
Operative time (h)	2.62 ± 0.57	2.42 ± 0.41	2.78 ± 0.29	2.37 ± 0.36	0.263

Data are presented as mean ± standard deviation or mean (interquartile range); ^*^ASA, American Society of Anesthesiologist physical status classification system, range 1 (normal health) to 5 (moribund).

**Table 2:** MMSE scores in the 4 groups

**Group**	**Time**			
	**D0**	**D1**	**D2**	**D3**
Control	28.7 ± 2.1	22.6 ± 2.9^*^	24.1 ± 3.2^*^	24.3 ± 2.8^*^
S	27.6 ± 3.2	23.7 ± 2.6^*^	24.8 ± 3.4^*^	27.1 ± 3.5^*^
M	28.0 ± 1.7	27.3 ± 2.2^#^	27.7 ± 3.1^#^	28.2 ± 3.3^#^
L	28.4 ± 2.6	27.8 ± 1.7^#^	28.2 ± 2.5^#^	28.1 ± 2.7^#^

Data are reported as mean ± standard deviation. MMSE, Mini Mental‑State Examination; D0: prior to induction of anesthesia (baseline); D1: 1 day after surgery; D2: 3 days after surgery; D3: 7 days after surgery. ^*^P < 0.05 vs before surgery; ^#^*P *< 0.05 *vs.* control group.

**Table 3.** Incidence of POCD in the four groups after surgery (n, %).

**Group**	**Time**		
	**D1**	**D2**	**D3**
Control	13 (43.3)	12(40.0)	12(40.0)
S	11(36.7)	10(33.3)	11(36.7)
M	5(16.7)^*^	5(16.7)^*^	4(13.3)^*^
L	3(10.0)^#^	2(6.7)^#^	2(6.7)^#^

n, % is number with the characteristic and percentage; D1: 1 day after surgery; D2: 3 days after surgery; D3: 7 days after surgery. ^*^P < 0.05 *vs.* control group; ^#^*P *< 0.01 *vs.* control group.

**Table 4:** Incidence of adverse effects and recovery time of the patients in the 4 groups

**Group**	**Adverse effect (n, %)**		**Recoverytime (min)**
	**Bradycardia Hypotension**		
Control	7(23.3)	8(26.7)	18.2 ± 2.7
S	8(26.7)	11(36.6)	15.3 ± 2.2
M	12(40.0)	10(33.3)	16.7 ± 3.4
L	20(66.7)^*#^	21(70.0)^*#^	27.3 ± 3.8^#^

n, % is number with the characteristic and percentage ;^ *^P < 0.05 *vs.* control group ; ^#^*P *< 0.05 *vs.* group M.

## Conclusion

Dexmedetomidine with a loading dose of 0.3 mcg/kg followed by maintenance doses of 0.5 and 0.8 mcg/kg/h (preferentially 0.5 mcg/kg/h) can reduce the incidence of POCD in elderly patients undergoing spine surgery. Dexmedetomidine reduces the incidence of POCD by inhibits the secretion of Aβ and proinflammatory cytokines,down-regulating the inflammatory response. Dexmedetomidine can be safely applied during anesthesia in elderly patients to prevent POCD.
